# Tofacitinib restores the balance of γδTreg/γδT17 cells in rheumatoid arthritis by inhibiting the NLRP3 inflammasome

**DOI:** 10.7150/thno.47860

**Published:** 2021-01-01

**Authors:** Xinyu Yang, Ning Zhan, Yang Jin, Hanzhi Ling, Chipeng Xiao, Zhen Xie, Hao Zhong, Xinxin Yu, Runhua Tang, Jinglan Ma, Jubo Guan, Guoyu Yin, Gan Wu, Liangjing Lu, Jianguang Wang

**Affiliations:** 1 Department of Biochemistry, School of Basic Medical Sciences, Wenzhou Medical University, Wenzhou, China.; 2 Department of Medicinal Chemistry, School of Pharmaceutical Sciences, Wenzhou Medical University, Wenzhou, China.; 3 School of Clinical Medicine, Hangzhou Medical College, Hangzhou, China.; 4 Department of Rheumatology, Shanghai Institute of Rheumatology, Renji Hospital, Shanghai Jiao Tong University School of Medicine, Shanghai, China.

**Keywords:** Rheumatoid arthritis, Tofacitinib, NLRP3 inflammasome, γδT cells, Inflammation

## Abstract

**Objective:** Tofacitinib (TOF) is a Janus kinase (JAK) inhibitor used in the treatment of rheumatoid arthritis (RA), but the mechanism of its action remains unclear. In this study, we investigated the influence of TOF on gamma delta regulatory T-cell (γδTreg)/γδT17 cell balance in RA and the role of the nucleotide-binding domain (NOD)-like receptor protein 3 (NLRP3) inflammasome in this process.

**Methods:** We detected levels of inflammatory factors in the serum of RA patients before and after administration of TOF using an enzyme-linked immunosorbent assay (ELISA). A collagen-induced arthritis (CIA) model was constructed to investigate the effect of TOF on arthritis symptoms, γδTreg/γδT17 cell balance and the NLRP3 inflammasome. We used bone marrow-derived macrophages (BMDMs) to study the effect of TOF on NLRP3 inflammasome activation. *Nlrp*3^-/-^ mice were introduced to assess the influence of NLRP3 on γδT17 cell activation in RA.

**Results:** TOF treatment decreased levels of γδT17 cell-related cytokine interleukin-17 (IL-17) in RA patients. In addition, TOF intervention in the CIA model reduced joint inflammation and damage, rebalanced the γδTreg/γδT17 cell ratio and inhibited excessive NLRP3 inflammasome activation in draining lymph nodes and arthritic joints. BMDM intervention experiments demonstrated that TOF decreased the level of secreted IL-1β via downregulation of NLRP3. Furthermore, experiments using *Nlrp3*^-/-^ mice verified that the NLRP3 inflammasome mediated the effect of TOF on γδT17 cell activation.

**Conclusions:** Recovery of γδTreg/γδT17 cell balance was a novel mechanism by which TOF alleviated RA. Meanwhile, NLRP3 played a pivotal role in the process of TOF-mediated γδT17 cell activation.

## Introduction

Rheumatoid arthritis (RA) is a very common chronic autoimmune disease characterized by inflammation and joint destruction that can well lead to substantial disability 1-3. Although the precise pathogenesis of RA remains unclear, the majority of available evidence points out that one pathology in RA is induced by IL-17 signaling and that IL-17 expression is increased during RA 4, 5. In addition, several recent studies have demonstrated that IL-17-producing γδ T-helper 17 (γδT17) cells are a major innate cellular source of IL-17 in collagen-induced arthritis (CIA) models 6-8. γδT17 cells are present in the synovium of mice in numbers equal to those of regular T-helper 17 (T_h_17) cells, and their proportion in the joints rises more dramatically than that of T_h_17 cells when mice develop CIA 9. Furthermore, γδT cell counts are increased in the synovium of patients with RA 10, 11. In contrast, Xia Yang has demonstrated that γδ regulatory T cells (γδTregs) mediate the anti-inflammatory response in asthma 12. Relative amounts of γδT17 cells and γδTregs in joints with or without inflammation have not yet been confirmed. Considering all of the above, we felt obliged to further investigate the influence of γδTreg/γδT17 cell disequilibrium in RA, which is a potential therapeutic target in the disease.

Tofacitinib (TOF) is a well-known small-molecule Janus kinase (JAK) inhibitor that preferentially targets JAK1 and JAK3 and, to a lesser extent, JAK2 and tyrosine kinase 2 (TYK2). Although the drug is recommended for the treatment of clinically active RA, the specific effect of TOF on critical immune events in RA pathogenesis, such as the restoration of lymphocyte equilibrium, remains unclear 13-15. Gao W verified that TOF can efficiently suppress IL-17 production in synovia, but it is not clear which cell type produces IL-17 16. Considering that γδT17 cells are a major source of IL-17 in arthritic joints, that TOF's mechanism of action is vague, and that no research on γδTregs in RA has been performed, how TOF restores the balance of γδTregs/γδT17 cells and the underlying mechanism by which this happens are pending confirmation.

The nucleotide-binding domain (NOD)-like receptor protein 3 (NLRP3) inflammasome is a multi-protein complex that is composed of NLRP3, apoptosis-associated speck-like protein containing a caspase activation and recruitment domain (ASC) and Caspase-1 (CASP-1). It is expressed on various types of cells, including macrophages and peripheral-blood leukocytes 17-19. Recent studies have demonstrated the potential role of NLRP3 in RA; increased activation of the NLRP3 inflammasome has been observed in swollen joints of CIA mice (a recognized model of RA), but *Nlrp*3 knockout mice have never been used for CIA modeling and TOF treatment experiments. In addition, Furuya MY recently confirmed that TOF might exert its therapeutic role by regulating NLRP3 in neutrophils 20. Simultaneously, Billon demonstrated that the NLRP3 inflammasome is a component of the innate immune system that leads to the processing and secretion of mature IL-1β, which plays a critical role in activating γδT17 cells to secret IL-17 21-23. These facts indicate a potential relationship between NLRP3 and the activation of γδT17 cells, which calls for further study to establish the functional mechanisms involved.

In this study, we investigated whether TOF regulated the balance of γδTregs/γδT17 cells during the pathogenesis of RA and examined the role of the NLRP3 inflammasome in the therapeutic mechanism.

## Methods and Materials

### Patients and samples

We obtained serum and synovia from RA patients and healthy controls (HCs) during joint arthroplasty with femoral-neck fracture. Peripheral-blood samples were acquired from patients with RA before and after treatment with oral TOF (5 mg twice daily for 12 weeks). All samples were collected during the initial visits of RA patients who had received no other medical treatment before diagnosis at the First Affiliated Hospital of Wenzhou Medical University, Wenzhou, China, and the Central Hospital of Jiamusi City, China. RA patients fulfilled 2010 American College of Rheumatology (ACR) criteria. All participants signed informed consent. Detailed clinical information is provided in Table S1.

### ELISA

We detected levels of IL-1β, IL-18, IL-17A, IL-6, IL-10, tumor necrosis factor alpha (TNF-α) and transforming growth factor beta (TGF-β) in serum and synovia using an enzyme-linked immunosorbent assay (ELISA) 24. The antibodies of cytokines mentioned above were purchased from BioLegend (San Diego, California, US). Specimens were diluted to 50 μL (1:20) per manufacturer's instructions and measured at an optical density (OD) of 450 nm.

### Western blot

We obtained lysates and separated proteins using sodium dodecyl sulfate polyacrylamide gel electrophoresis (SDS-PAGE) and subsequently transferred them to polyvinylidene difluoride (PVDF) membranes. Blots were incubated with primary antibodies (anti-NLRP3 [AdipoGen Life Sciences, San Diego, California, US], anti-CASP-1 [R&D Systems, Inc., Minneapolis, Minnesota, US], anti-IL-1β [Santa Cruz Biotechnology, Dallas, Texas, US] or β-tubulin [bioWORLD, Dublin, Ohio, US]) overnight at 4°C. Incubation with secondary anti-rabbit horseradish peroxidase (HRP) or anti-goat HRP (Hangzhou MultiSciences [Lianke] Biotech Co., Ltd., Hangzhou, China) was performed at room temperature (RT) for 1 h. We used an Electrochemiluminescence (ECL) Plus Western Blot Detection Kit (Amersham Biosciences [GE Healthcare, Chicago, Illinois, US]) for antibody detection.

### Mice, collagen-induced arthritis model construction and clinical evaluation

We purchased 8-week-old male Dilute, Brown, and non-Agouti/1 (DBA/1 mice (20-22 g in body weight [BW]) from Shanghai Laboratory Animal Center (SLAC) and *Nlrp*3^-/-^ mice from Zhejiang Laboratory Animal Center. All mice were raised in a specific-pathogen-free room in the Laboratory Animal Center of Wenzhou Medical University, housed five to a cage and kept at 22-26°C with 60%-65% humidity on a regular 12-h light and dark cycle (light period, 8:30-20:30). Standard laboratory chow and water were available *ad libitum*. We performed experiments under pathogen-free conditions, and personnel tending the mice regularly examined their health status. No adverse events were observed. All animal experiments were endorsed by the Institutional Animal Care and Use Committee of Wenzhou Medical University.

Mice were randomly assigned into groups of 10. We induced autoimmune arthritis in the mice under anesthesia (xylazine, 5-10 mg/kg [BW] intraperitoneally [i.p.]; and ketamine, 50 mg/kg BW i.p.) by injecting a 100 μL emulsion of complete Freund's adjuvant and 2 mg/mL type II bovine collagen (both Chondrex, Redmond, Washington, US) intradermally at the base of the tail on day 0. Three weeks later, we gave the mice a booster immunization in a 100 μL emulsion in which type II bovine collagen (2 mg/mL) was emulsified with incomplete Freund's adjuvant. Mice were then given TOF orally (0 or 120 ng) daily from day 21 (arthritis induction) to day 48, or from day 36 (emergence of noticeable arthritis symptoms) to day 48 (Figs. 2A, 4A). After the second immunization, two independent observers who were not aware of the animals' treatment inspected mice every 3 days for the severity of arthritis. Mice were euthanized on day 49. Our scoring system was as follows: 0 = no evidence of erythema or swelling; 1 = erythema and mild swelling confined to the tarsals or ankle joint; 2 = erythema and mild swelling extending from the ankle to the tarsals; 3 = erythema and moderate swelling extending from the ankle to the metatarsal joints; and 4 = erythema and severe swelling encompassing the ankle, paws and digits, or ankylosis of the limb. We gathered joint tissues, immune organs and serum for further study. The limbs were fixed in 4% paraformaldehyde, decalcified in 5×10^-5^ mM ethylenediaminetetraacetic acid (EDTA) solution and embedded in paraffin 25.

### Immunohistochemistry (IHC)

Formalin-fixed paraffin sections of joints were subjected to rehydration, and endogenous peroxidase activity was quenched with 3% H_2_O_2_. Next, we blocked the sections with bovine serum albumin (BSA) and incubated them with primary anti-IL-1β (Santa Cruz Biotech) per manufacturer's protocol. Slides were incubated with HRP-conjugated secondary antibody (Abcam, Cambridge, UK), visualized using the 3,3'-Diaminobenzidine (DAB) technique, counterstained with hematoxylin and dehydrated. We evaluated the sections using an Eclipse 80i microscope and analyzed images using Nikon NIS-Elements software (both Nikon, Tokyo, Japan).

### Immunofluorescence (IF)

We blocked paraffin sections with BSA and then incubated them with primary antibodies (anti-TCRγ/δ, anti-IL-17 or anti-*FoxP3*) overnight at 4°C in a humidified chamber. Slides were then incubated with fluorescein isothiocyanate (FITC)-conjugated secondary antibodies for 1 h at RT in the dark. Nuclei were counterstained with 4′,6-diamidino-2-phenylindole (DAPI). All antibodies involved in this experiment were purchased from Santa Cruz Biotech. We used C2 PLUS confocal microscopy (Nikon) for imaging.

### Ribonucleic acid (RNA) isolation and quantitative reverse-transcription polymerase chain reaction (RT-qPCR)

Total RNA samples were isolated from murine tissue or cells using TRIzol reagent (Ambion, Inc., Austin, Texas, US) per manufacturer's protocol. We reverse-transcribed 1 μg template RNA using a PrimeScript RT reagent kit (TaKaRa Bio, Shiga, Japan) and measured expression of the target gene using a SYBR Fast Universal qPCR Kit (Kapa Biosystems, Inc. [Roche Life Science, Basel, Switzerland]) for real-time qPCR. The following primer sequences were used: NLRP3: F, AGA TTA CCC GCC CGA GAA AG; R, TCC CAG CAA ACC CAT CCA CT; and β-actin: F, CCT TCC TTC TTG GGT ATG GA; R: ACG GAT GTC AAC GTC ACA CT. We normalized the final calculated results to β-actin and converted them using relative quantification (2^-ΔΔCt^).

### Flow cytometry (FCM)

We gently ground the tissues with 200-mesh cell strainers, washed them twice in phosphate-buffered saline (PBS) and resuspended them in Roswell Park Memorial Institute (RPMI) 1640 medium. Cells were stained with APC-conjugated anti-CD3 and then FITC-conjugated anti-gamma delta T-cell receptor (anti-TCRγ/δ) at 4°C in the dark. After surface staining, we stimulated γδT17 cells with Leukocyte Activation Cocktail (1 μL/mL) for 4 h at 37°C in 5% CO_2_. We then labeled γδT17 cells and γδTregs with phycoerythrin (PE)-conjugated anti-IL-17 and PE-conjugated anti-*FoxP3*, respectively. FCM was performed using a Cytoflex flow cytometer and analyzed using FlowJo™ v10.7 software (both BD Biosciences, Franklin Lakes, New Jersey, US). All reagents involved in this experiment were purchased from BD Biosciences.

### Bone marrow-derived macrophage isolation and stimulation

Using RPMI 1640 medium, we rinsed bone marrow cells from the bilateral hind femurs of DBA/1 mice. We cultured 1×10^6^ BMDMs in RPMI 1640 medium supplemented with 10% heat-inactivated fetal bovine serum (FBS), 2 mM L-glutamine and 20% conditioned L929 medium in a 12-well plate for 6 days. Adherent macrophages were harvested and stimulated with lipopolysaccharide (LPS) for 6 h and with nigericin for 2 h to activate the NLRP3 inflammasome *in vitro*. Then, we treated the macrophages with TOF (1, 5 or 25 × 10^-3^ mM) for 2 h.

### Statistical analysis

We used SPSS software version 22.0 (IBM Corp., Armonk, New York, US) and GraphPad Prism version 8 (GraphPad Software, Inc., San Diego, California, US) for statistical analysis. Normally distributed data were judged using the Shapiro-Wilk method. The Levene method was used to test homogeneity of variance. Two sets of data that met normal distribution and homogeneity of variance were analyzed using Student's *t* test. Multigroup comparisons of means were carried out by a one-way analysis of variance (ANOVA) test, with *post hoc* contrasts performed using Tukey's test. Data that did not meet homogeneity of variance or normal distribution were compared using Kruskal-Wallis and Mann-Whitney non-parametric tests. *P*-values < 0.05 were considered significant.

## Results

### NLRP3 inflammasome was overactivated, and the γδTreg/γδT17 cell ratio decreased in RA patients

**Figure 1:**
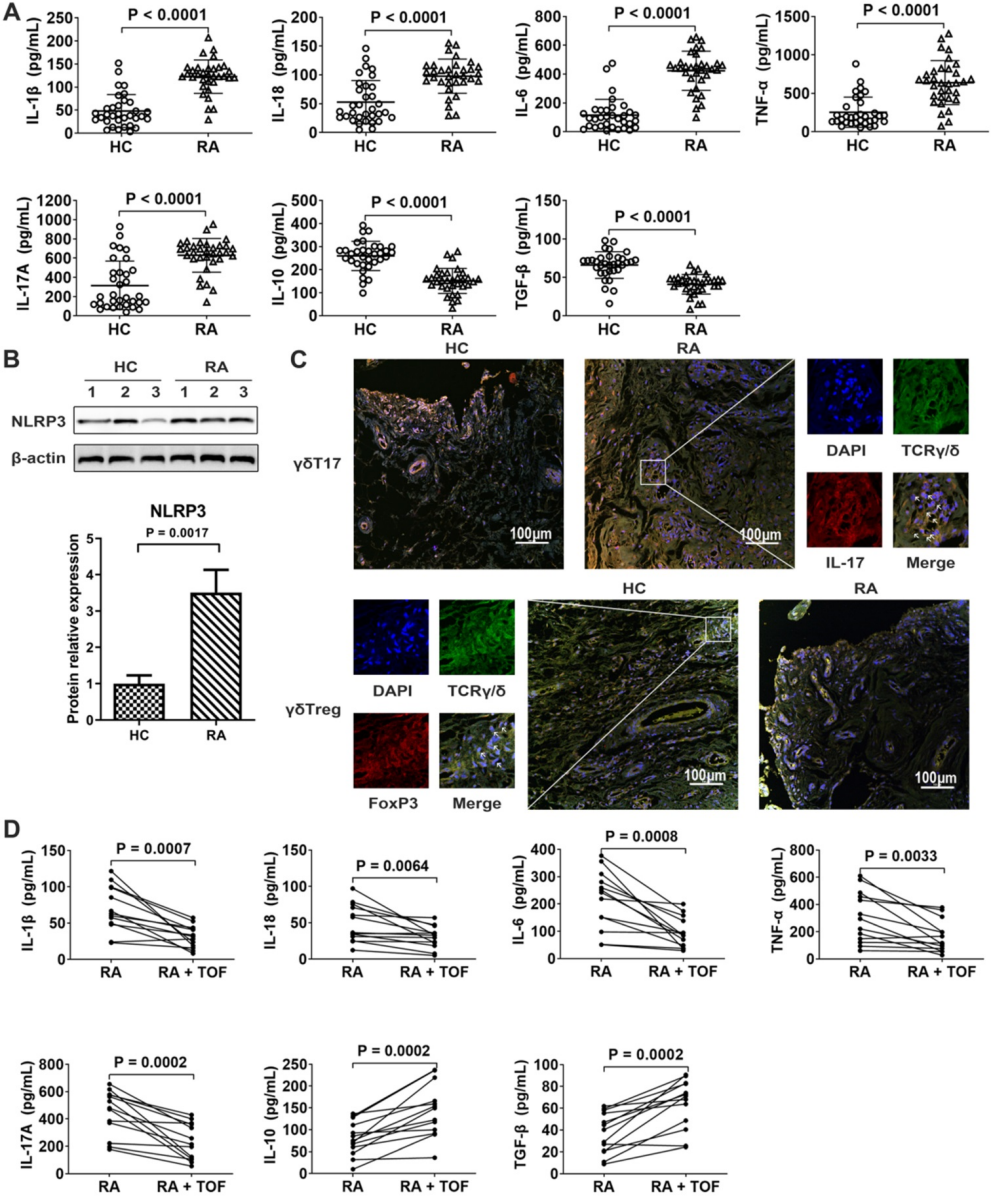
** TOF treatment inhibited NLRP3 inflammasome levels and balanced the γδlang/γδT17 ratio in rheumatoid arthritis (RA).** (**A**) Levels of cytokines IL-1β, IL-18, IL-6, TNF-α, IL-17, IL-10 and TGF-β in synovia of RA patients (n = 33) and HCs (n = 33) were detected by ELISA. (**B**) Immunoblot analysis of NLRP3 in synovial lysates of RA patients (n = 10) and HCs (n = 10). (**C**) Representative images of paraffin sections from RA patients (n = 10) and HCs (n = 10) that were stained with anti-human TCRγ/δ (green) and anti-human IL-17 (upper, red) or with anti-human TCRγ/δ (green) and anti-human *Foxp3* (lower, red) for IF analyses. Arrows in the merged image indicate γδT17 cells (upper) or γδTregs (lower). IL-17-positive but TCRγ/δ-negative staining areas of the merged images represent other IL-17-secreting cells. One of the 10 independent experiments is shown. (**D**) Levels of IL-1β, IL-18, IL-17, IL-6, TNF-α, IL-10 and TGF-β in serum of RA patients (n = 13) and TOF-treated patients (n = 13) were detected by ELISA.

NLRP3 inflammasome-dependent cytokines IL-1β and IL-18 were higher in RA synovia than in those of HCs (Fig. 1A). Accordingly, the protein level of NLRP3 increased in the synovium of RA patients (Fig. 1B). The γδT17 cell-related cytokine IL-17 increased in RA synovia (Fig. 1A). Compared with HCs, RA raised the percentage of γδT17 cells but decreased that of γδTregs in synovium (Fig. 1C). TOF treatment downregulated pro-inflammatory cytokines IL-1β, IL-18, IL-17, TNF-α and IL-6 while upregulating anti-inflammatory cytokines IL-10 and TGF-β, thus tending to restore the abovementioned serum cytokines levels to normal range (Fig. 1D).

### TOF intervention alleviated joint injury and inflammatory response in a CIA model

**Figure 2:**
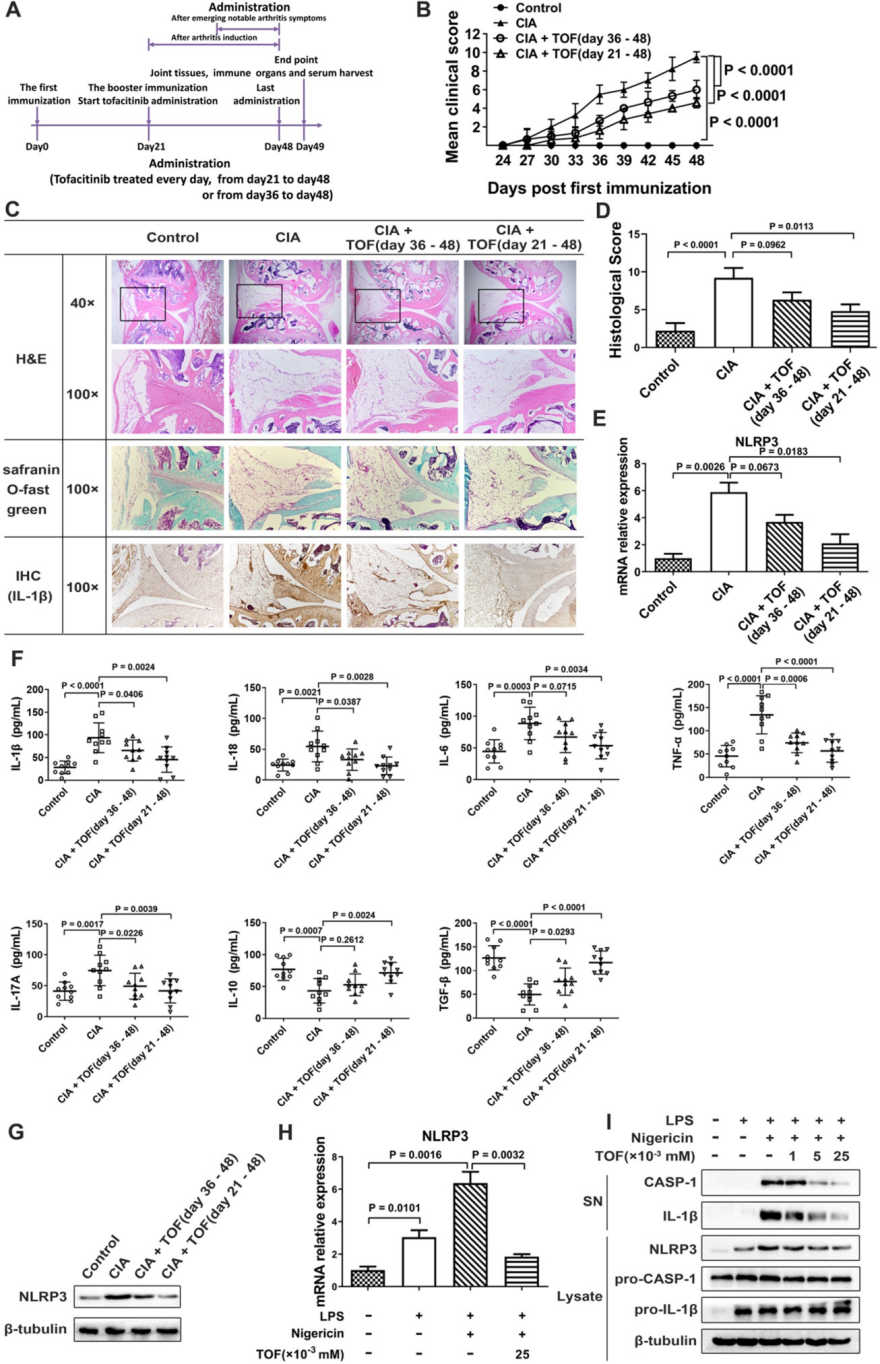
** TOF ameliorated joint inflammatory response and inhibited NLRP3 inflammasome activation in a CIA model.** (**A**) Timeline of TOF intervention experiment in the CIA model. (**B**) Clinical scores of CIA mice during TOF administration. Two independent observers who were not aware of the animals' treatment inspected the mice every 3 days for the severity of arthritis. Scoring was as follows: 0 = no evidence of erythema or swelling; 1 = erythema and mild swelling confined to the tarsals or ankle joint; 2 = erythema and mild swelling extending from the ankle to the tarsals; 3 = erythema and moderate swelling extending from the ankle to the metatarsal joints; and 4 = erythema and severe swelling encompassing the ankle, paw and digits, or ankylosis of the limb. Statistical significance was determined by ANOVA of repeated measurements. n = 10 per group. (**C**) Knee joints of CIA mice (n = 12) were stained with H&E (blue for cell nuclei and membranes; red for cytoplasm and extracellular matrix [ECM]) and Safranin O Fast Green (green for cartilages) and graded on a scale of 0 (normal) to 3 (severe). Expression of IL-1β in paraffin sections of synovia of CIA mice was detected by IHC analysis. (**D**) Histological score for CIA mice was F(3, 36) = 9.358. n = 10 per group. (**E**) Using RT-qPCR, expression of NLRP3 mRNA was primarily detected in cultured lymphocytes from lymph nodes of CIA mice treated with TOF (F^3^, ^36^ = 13.97). n = 10 per group. (**F**) Concentrations of IL-1β, IL-18, IL-6, TNF-α, IL-17, IL-10 and TGF-β in serum from CIA mice were detected by ELISA. Results were F(3, 36) = 12.19 for IL-1β, F(3, 36) = 6.971 for IL-18, F(3, 36) = 7.258 for IL-6, F(3, 36) = 19.26 for TNF-α, F(3, 36) = 6.016 for IL-17, F(3, 36) = 8.083 for IL-10 and F(3, 36) = 19.98 for TGF-β. n = 10 per group. (**G**) Protein levels of NLRP3 in lymph nodes of CIA mice treated with TOF were detected by Western blot. (H) LPS-primed BMDMs were stimulated with nigericin and then treated with TOF for 2 h. Expression of NLRP3 mRNA in BMDMs was detected by RT-qPCR (F^3^, ^36^ = 31.37). n = 10 per group. (I) Immunoblot analysis of IL-1β and cleaved CASP-1 (p20) in culture supernatants (SNs); immunoblot analysis of NLRP3, precursors of IL-1β (pro-IL-1β) and precursors of CASP-1 (pro-CASP-1) in lysates of BMDMs. n = 10 per group.

We used a CIA model to study the effect of TOF on the RA process. TOF intervention could reduce clinical score and paw swelling in CIA mice (Fig. 2B). Meanwhile, we detected a dose-dependent decrease in histological score after TOF treatment (Fig. 2C and D). NLRP3 inflammasome-dependent cytokines IL-1β and IL-18 were indeed inhibited by TOF in a dose-dependent manner (Fig. 2F). Moreover, the protein level of IL-1β decreased in synovium from TOF-treated mice (Fig. 2C). Simultaneously, NLRP3 expression declined in the lymph nodes of the TOF-treated group (Fig. 2E, G).

### TOF restored γδTreg/γδT17 cell balance

**Figure 3:**
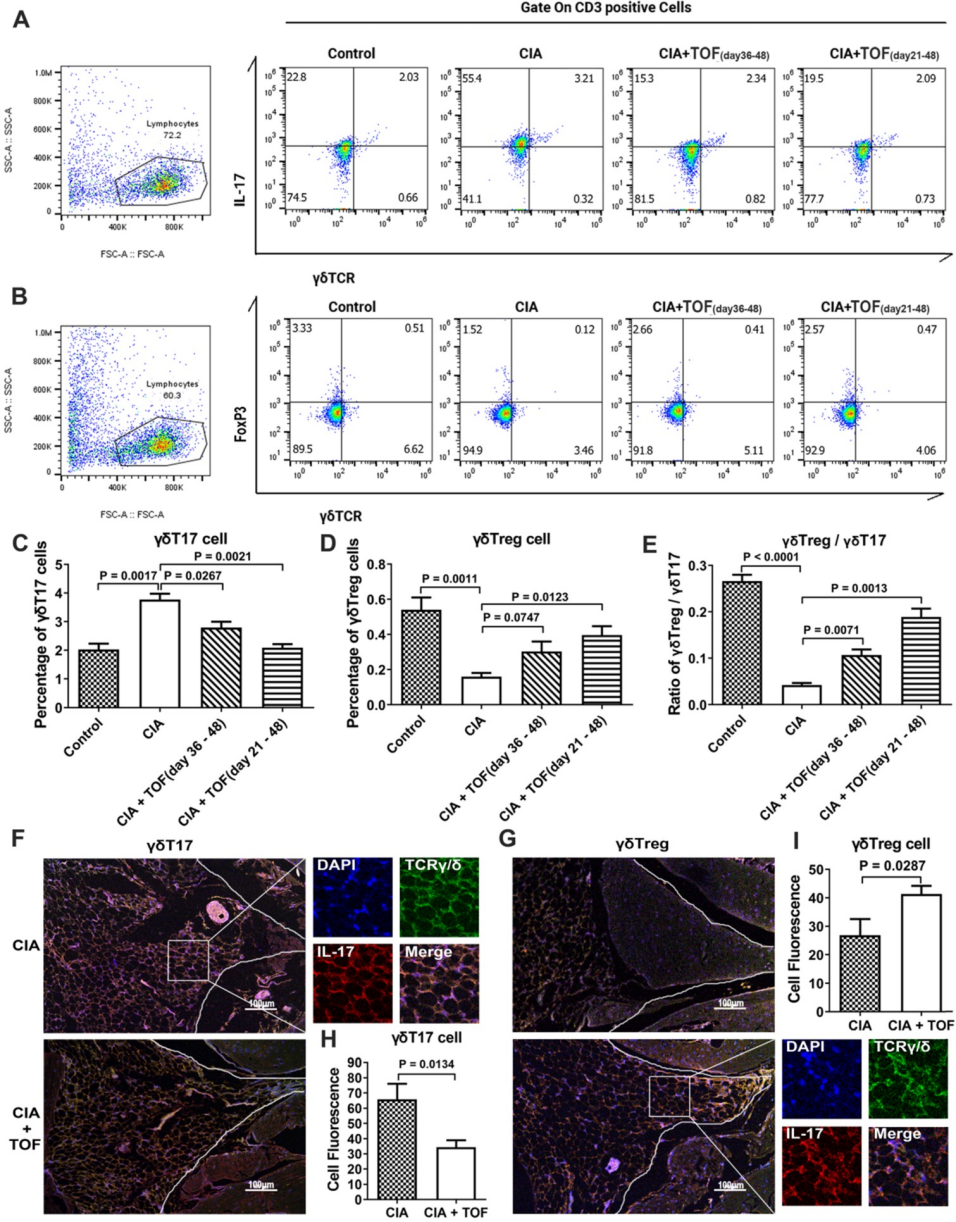
** TOF influenced γδTreg/γδT17 balance in CIA mice. (A, C)** Representative FCM images indicated percentages of γδT17 cells in lymph nodes of CIA mice treated with TOF (F^3^, ^36^ = 23.68). n = 10 per group. **(B, D)** Percentage of γδTregs was detected in lymph nodes of CIA mice treated with TOF (F(3, 36) = 13.16). n = 10 per group. **(E)** γδTreg/γδT17 cell ratio in lymph nodes of CIA mice treated with TOF (F^3^, ^36^ = 69.96). **(F-I)** Representative images of paraffin sections were stained with anti-mouse TCRγ/δ (green) and anti-mouse IL-17 (upper, red), or with anti-mouse TCRγ/δ (green) and anti-mouse *Foxp3* (lower, red) for IF analyses. Arrows in the merged image indicate γδT17 cells (upper) or γδTregs (lower). One of the 10 independent experiments is shown.

There has been no research into the efficacy of TOF on percentage of γδT17 cells versus that of γδTregs. Therefore, our finding that TOF decreased the levels of γδT17 cells and γδT17 cell-related cytokine IL-17 while increasing that of γδTregs in a dose-dependent manner (Figs. 2F, 3A-D) was novel. Consequently, the ratio of γδTregs/γδT17 cells was elevated in the TOF-treated group (Fig. 3E). IF results indicated that after TOF intervention, the percentage of γδT17 cells was reduced, while that of γδTregs was elevated in paraffin sections of knee joint samples (Fig. 3F-I). The γδTreg/γδT17 cell balance changed more significantly in mice that began treatment on day 21 instead of day 36.

### TOF inhibited excessive NLRP3 inflammasome activation

Levels of cleaved CASP-1 (p20) and inflammatory cytokine IL-1β decreased in BMDMs after TOF treatment, while expression of pro-CASP-1 and pro-IL-1β exhibited no significant changes (Fig. 2I). These results indicated that TOF inhibited the inflammasome effector molecule CASP-1 autocleavage and IL-1β secretion but did not downregulate the synthesis of their precursors. The NLRP3 inflammasome plays a vital role in the self-cleavage of pro-CASP-1 and generation of IL-1β. Messenger RNA (mRNA) levels of NLRP3 and protein expression levels of NLRP3 were markedly increased in LPS-induced BMDMs compared with the control group, whereas these levels were distinctly decreased by TOF treatment (Fig. 2H and I). These results indicated that TOF suppressed the NLRP3 inflammasome by inhibiting NLRP3 expression at the transcription level and then reducing cleaved CASP-1, ultimately decreasing levels of the mature cytokine IL-1β.

### TOF inhibited activation of γδT17 cells via suppression of the NLRP3 inflammasome

**Figure 4:**
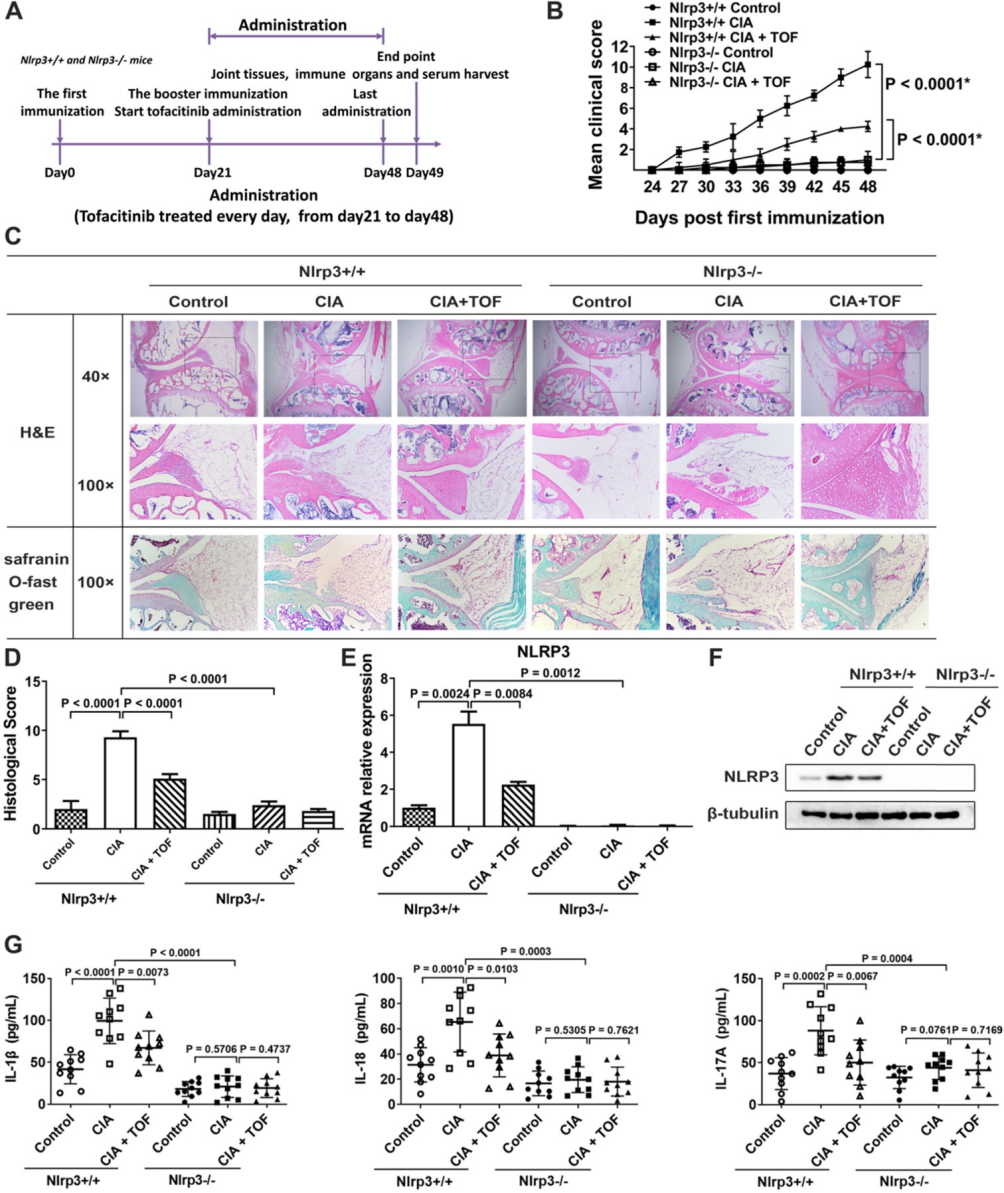
** TOF alleviated joint injury and inflammatory response via suppression of the NLRP3 inflammasome.** (**A**) Timeline of TOF treatment in *Nlrp*3^+/+^ and *Nlrp*3^-/-^ mice in CIA models. (**B**) Clinical scores of CIA models established from *Nlrp*3^+/+^ and *Nlrp*3^-/-^ mice. Two independent observers who were not aware of the animals' treatment inspected the mice every 3 days for the severity of arthritis. Statistical significance was determined by ANOVA of repeated measurements. *Data are compared with *Nlrp*3^+/+^ control group. n = 10 per group. (**C**) Knee joints of mice were stained with H&E and Safranin O Fast Green. (**D**) Histological score of *Nlrp*3^+/+^ and *Nlrp*3^-/-^ mice was F(5, 54) = 62.96. n = 10 per group. (**E**) Using RT-qPCR, expression of NLRP3 mRNA was primarily detected in cultured lymphocytes from lymph nodes of *Nlrp*3^+/+^ and *Nlrp*3^-/-^ mice (F[5, 54 = 60.10). n = 10 per group. (**F**) Protein levels of NLRP3 were detected in lymph nodes of *Nlrp*3^+/+^ and *Nlrp*3^-/-^ mice using Western blot. n = 10 per group. (**G**) Concentrations of IL-1β, IL-18 and IL-17 in serum from *Nlrp*3^+/+^ and *Nlrp*3^-/-^ mice were detected by ELISA. Results were F(5, 54) = 35.92 for IL-1β, F(5, 54) = 15.26 for IL-18 and F(5, 54) = 9.061 for IL-17. n = 10 per group.

**Figure 5:**
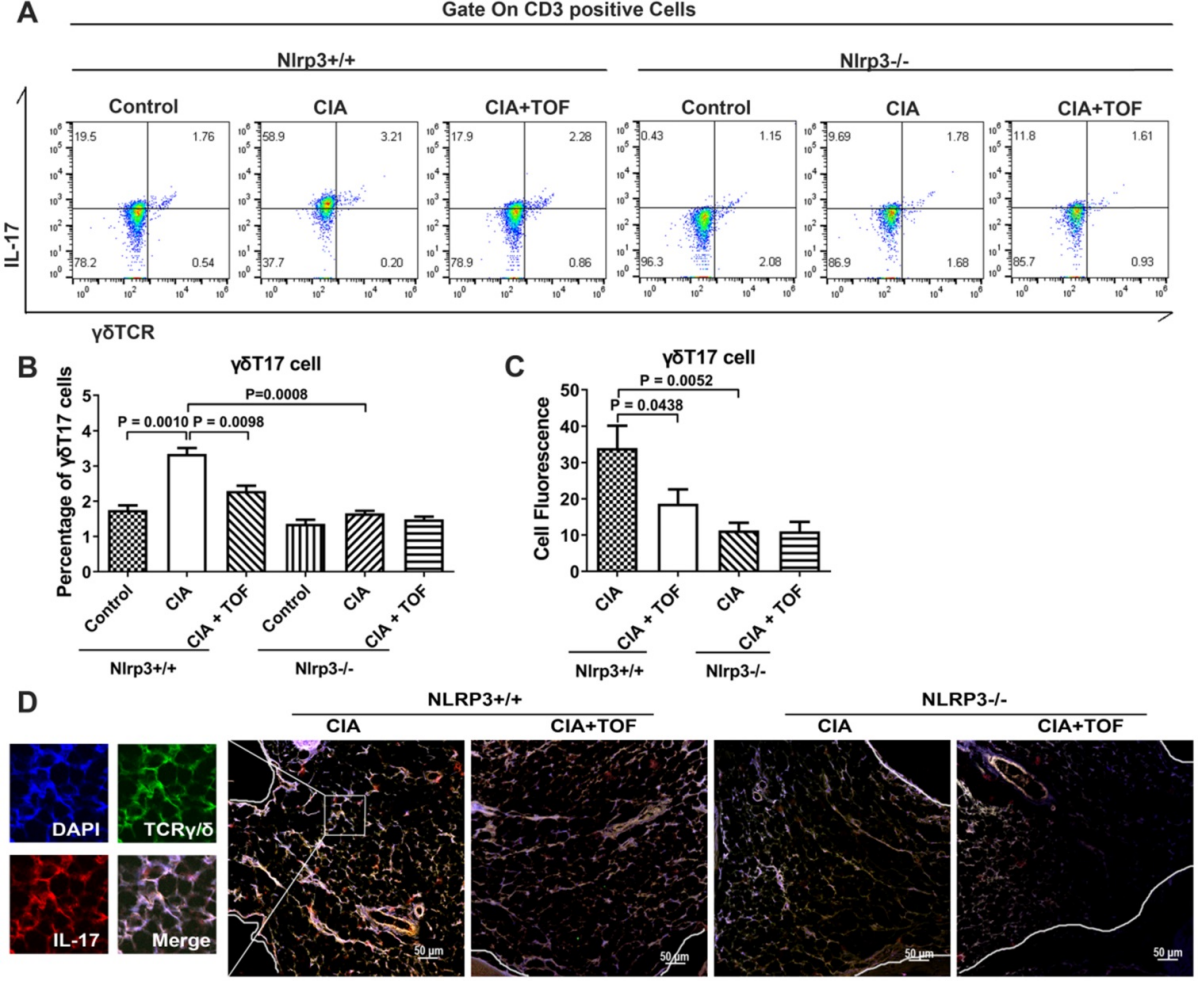
** TOF influenced the activation of γδT17 cells via suppression of the NLRP3 inflammasome.** (**A, B**) Representative FCM pictures indicated percentages of γδT17 cells in the lymph nodes of *Nlrp*3^+/+^ and *Nlrp*3^-/-^ mice (F[5,54] = 39.68). n = 10 per group. (**C, D**) Representative images of paraffin sections from *Nlrp*3^+/+^ and *Nlrp*3^-/-^ mice were stained with anti-mouse TCRγ/δ (green) and anti-mouse IL-17 (red) for IF analyses. Arrows in the merged image indicate γδT17 cells. One of the 10 independent experiments is shown (F^3^, ^36^ = 11.87).

To verify whether NLRP3 was a crucial downstream target of TOF in the treatment of RA, we innovatively constructed a CIA model using *Nlrp3*^-/-^ mice. However, we found no significant RA symptoms in these animals (Fig. 4B). TOF intervention significantly delayed the onset and reduced the severity of RA in *Nlrp3*^+/+^ CIA mice but had no effect on *Nlrp3*^-/-^ mice (Fig. 4B-D). At the same time, inflammatory factors in CIA mouse serum decreased after *Nlrp3* knockout (Fig. 4G). Expression of NLRP3 and IL-1β was abrogated in *Nlrp3* knockout tissues (Fig. 4C, E and F). We used FCM to analyze the proportions of γδT17 cells isolated from lymph nodes. Compared with control mice, TOF intervention reduced the percentage of γδT17 cells in *Nlrp*3^+/+^ mice rather than in *Nlrp3*^-/-^ mice (Fig. 5A and B). Meanwhile, when we observed IF staining in paraffin sections of knee joint samples from *Nlrp3*^+/+^ versus *Nlrp3*^-/-^ mice, percentages of γδT17 cells had changed to an obviously different extent (Fig. 5C and D). We also applied FCM and IF to detect γδTreg infiltration, but we saw no significant difference in the percentage of γδTregs between *Nlrp3*^+^/^+^ and *Nlrp3*^-^/^-^ mice after constructing our CIA model (Fig. S2). We further conducted experiments involving CIA DBA/1 mice treated with the NLRP3 inhibitor MCC950 (Fig. S1A). CIA mice given MCC950 in combination with TOF were more likely to express lower levels of the NLRP3 inflammasome than those given MCC950 alone (Fig. S1C and D). Arthritic symptoms were ameliorated more significantly, and activation of γδT17 cells was also restrained at lower levels, in CIA mice treated with both MCC950 and TOF than in mice treated with MCC950 alone (Fig. S1B, E and F).

## Discussion

RA is a chronic autoimmune disease that causes progressive articular damage and functional loss. Though its precise pathogenesis remains unclear, it has been reported that γδT17 cells are recruited by chemokine (C-C motif) ligand 20 (CCL20) expressed in vascular endothelial cells and fibroblast-like synoviocytes in inflamed joints and that they produce cytokines in immediate response to initial inflammatory stimulation 26-28. Considering that the role of γδTregs in RA has not yet been demonstrated, we detected not only the percentage of γδT17 cells but also that of γδTregs in synovium using IF. The results revealed that the percentage of γδT17 cells in RA patients was higher than in HCs, while γδTregs showed an inverse trend. In addition, the γδT17 cell-related cytokine IL-17 increased in the synovia of RA patients. However, as γδT17 cells were not recruited in peripheral blood, FCM results revealed a very low percentage thereof (data not shown). All of the above facts showed that γδT17 cells and γδTregs were apt to play significant roles in RA progression. Nonetheless, Yoshinaga demonstrated that in late-stage RA patients taking long-term medication, activities and the numbers of γδT17 cells decrease toward basal level in burned-out tissue. In this case, there might be other sources of IL-17 in arthritic joints, such as T_h_1 cells 29. However, using IF, we detected γδT17 cells above the basal level in synovium during initial visits by patients who had not received any other medical treatment before diagnosis. It is worth noting but not mutually exclusive that γδT17 cells are still inclined to predominate in the production of IL-17 during the early stage of synovial inflammation in RA.

TOF is an oral JAK inhibitor that is currently in clinical use for RA treatment, with promising results to date, but its specific mechanism has yet to be completely elucidated 13-15. We conducted a clinical experiment in which we administered the recommended dose of TOF to RA patients included in this study. We assessed changes in serum cytokines in these patients after TOF treatment, revealing that they had decreased from baseline. Meanwhile, the level of NLRP3 in synovium as detected by Western blot was higher in RA patients than in HCs. Accordingly, IL-1β and IL-18 were decreased in the serum of RA patients after TOF treatment; these cytokines can be produced through the NLRP3 inflammasome pathway. Therefore, we wanted to explore whether the therapeutic effect of TOF in RA progression was related to γδTreg/γδT17 cell balance and the NLRP3 inflammasome *in vivo*.

We constructed a CIA model to verify the effect of TOF on RA pathogenesis and γδTreg/γδT17 cell balance. First, hematoxylin and eosin (H&E) and Safranin O Fast Green staining demonstrated that TOF could alleviate inflammatory-cell infiltration, synovium proliferation and induced cartilage and bone damage in CIA. In addition, TOF downregulated IL-17 and restored γδTreg/γδT17 cell balance. Interestingly, mice given TOF immediately after arthritis induction showed better efficacy and more low-level activation of γδT17 cells than mice given TOF after noticeable arthritis symptoms had already emerged. This might be related to γδT17 cells being the initial source of IL-17 after inflammatory stimulation 28. At the same time, TOF intervention decreased IL-1β levels and repressed NLRP3 activation, but the role of NLRP3 in TOF's regulation of γδTreg/γδT17 cell balance requires further study.

The NLRP3 inflammasome is a multi-protein complex that contains NLRP3 and the adaptor ASC, which serve as platforms for autocleavage of the effector CASP-1 17, 30-32. Previous studies have found that selective NLRP3 inhibitor MCC950 was effective in ameliorating arthritic symptoms in CIA mice, indicating that NLRP3 might be involved in RA pathogenesis 33-35. In this study, we detected the effect of TOF on NLRP3 inflammasome activation in BMDMs. Results revealed that TOF downregulated the expression of NLRP3, thus inhibiting the release of IL-1β. Furthermore, it is well known that TOF is a small-molecule JAK inhibitor. JAK recruits and phosphorylates downstream signal transducers and activators of transcription (STATs); the phosphorylated STATs form dimers, translocate into the nuclei and regulate gene transcription 23, 30, 36, 37. Notably, Furuya *et al.* reported that TOF diminished granulocyte-macrophage colony-stimulating factor (GM-CSF)-stimulated NLRP3 protein expression and IL-1β secretion by inhibiting phosphorylation of STAT3/5 20. In dorsal-root ganglia, STAT3 significantly prevented acetylation of upregulated H3 and H4 in the NLRP3 promoter region, thus upregulating NLRP3 expression 38. These findings suggest that TOF might play some roles in the expression of NLRP3 through the JAK/STAT signaling pathway. Having determined these points, we investigated whether NLRP3 was a crucial node in the process of TOF-regulated γδT17 cell activation.

As mature IL-1β is secreted mainly by the NLRP3 inflammasome and plays a critical role in the activation of γδT17 cells to secrete IL-17 22, 23, 39, we conducted further experiments involving NLRP3 knockout mice and MCC950-treated mice to explore the effect of NLRP3 on γδT17 cell activation and how TOF influenced this process. Compared with control mice, TOF inhibited the activation of γδT17 cells in *Nlrp*3^+/+^ mice but had no significant effect on *Nlrp*3^-/-^ mice. Furthermore, CIA mice given MCC950 in combination with TOF were more likely to show a lower level of γδT17 cell activation than those given MCC950 alone. All the above facts indicated that TOF could inhibit the activation of γδT17 cells by suppressing the function of NLRP3.

In summary, we confirmed that TOF could effectively ameliorate RA progression by rehabilitating γδTreg/γδT17 cell balance and that NLRP3 played a pivotal role in the process of TOF-mediated γδT17 cell activation.

## Supplementary Material

Supplementary figures and tables.
