# Supplementary material for: Tofacitinib restores the balance of γδTreg/γδT17 cells in rheumatoid arthritis by inhibiting the NLRP3 inflammasome
Source: Theranostics. 2021 Jan 1;11(3):1446–57. doi: 10.7150/thno.47860 (PMC7738886; doi:10.7150/thno.47860)
Supplement: Supplementary file 1 — Supplementary figures and tables. [file thnov11p1446s1.pdf]

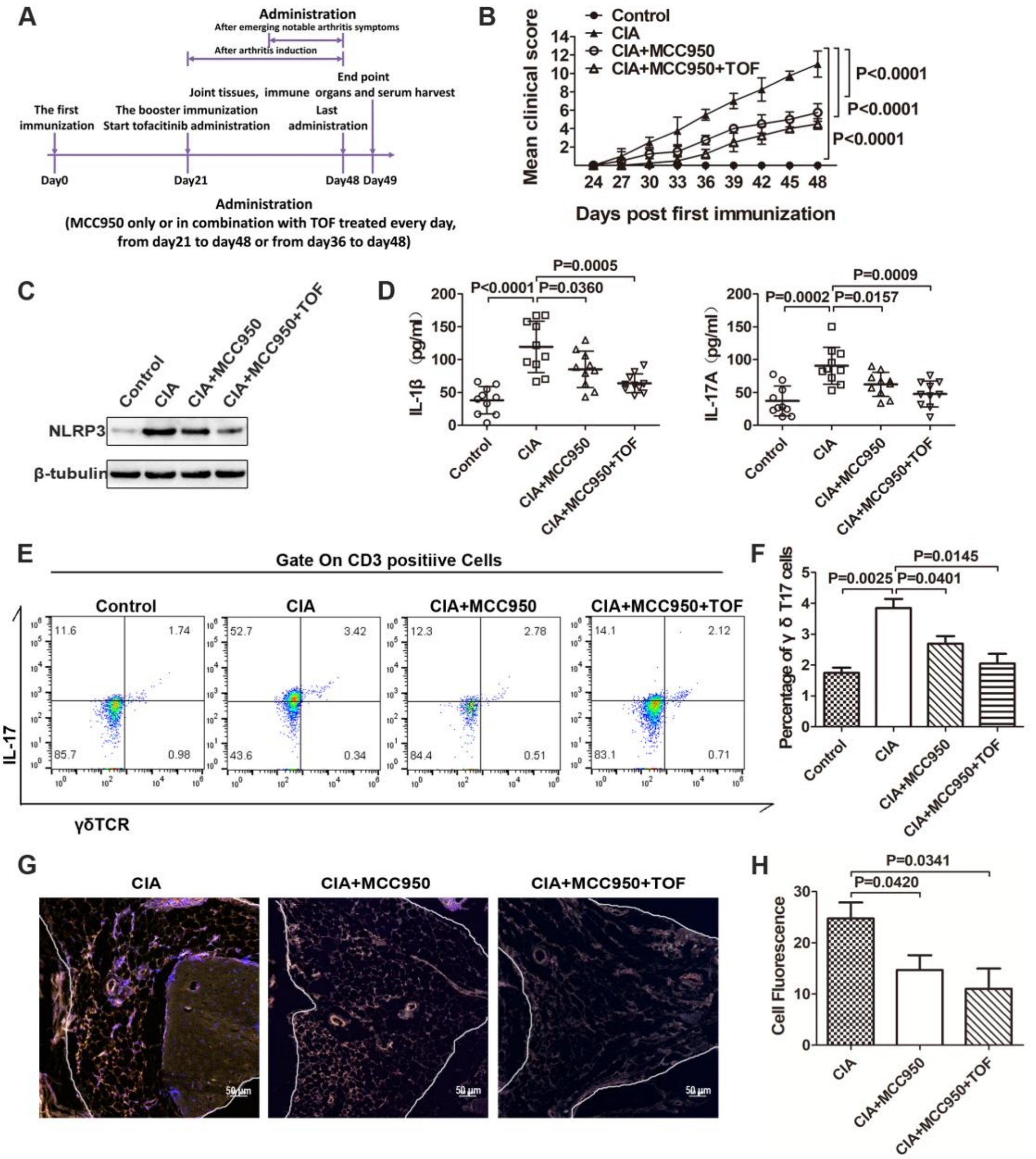

Figure S1. TOF ameliorated joint inflammatory response and influenced the activation of γδT17 cells in CIA model treated with MCC950. (A) Timeline of MCC950 and TOF intervention experiment in the CIA model. (B) Clinical scores of CIA mice during the MCC950 and TOF administration. Mice were inspected every three days for severity of arthritis by two independent observers who were not aware of the animal's treatment. Statistical significance was conducted by analysis of variance (ANOVA) of repeated measurement. n=10 per group. (C) The protein level of NLRP3 in lymph nodes of CIA mice treated with MCC950 and TOF was detected by western blot. (D) The concentration of IL-1β and IL-17 in serum from CIA mice was detected by ELISA ($F(3, 36)=16.15$ for IL-1β and $F(3, 36)=10.64$ for IL-17). n=10 per group. (E, F) Representative flow cytometric pictures indicated the percentage of γδT17 cells in lymph nodes of CIA mice treated with MCC950 and TOF ($F(3, 36)=13.31$). n=10 per group. (G, H) Representative images of paraffin sections were stained with anti-mouse TCRγ/δ (green) and anti-mouse IL-17 (red) to perform immunofluorescence analyses. Arrows in the merged image indicate γδT17 cells. One of the ten independent experiments is shown ($F(2, 27)=5.56$).

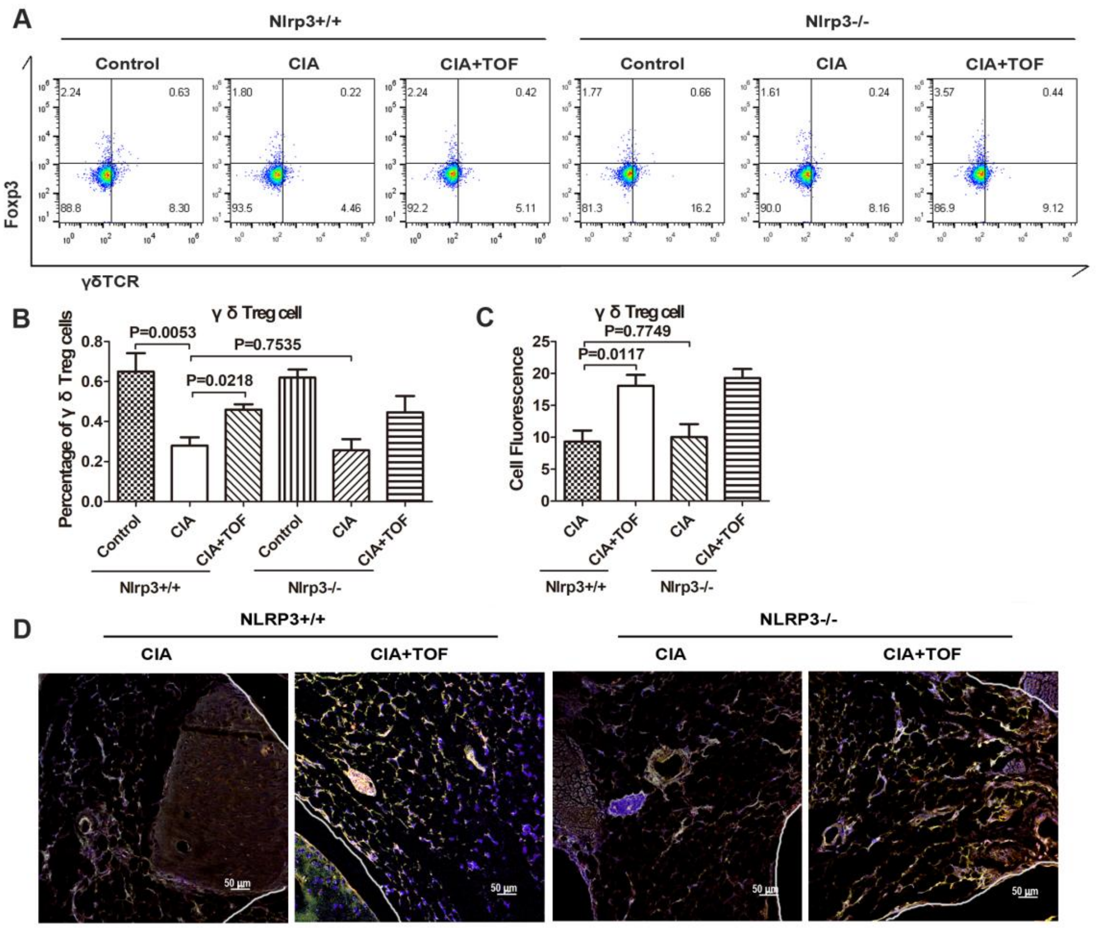

Figure S2. TOF influenced the percentage of γδTreg cells but not via suppression of NLRP3 inflammasome. (A, B) Representative flow cytometric pictures indicated the percentage of γδTreg cells in lymph nodes of Nlrp3$^{+/+}$ and Nlrp3$^{-/-}$ mice (F(5, 54)=9.86). n=10 per group. (C, D) Representative images of paraffin sections from Nlrp3$^{+/+}$ and Nlrp3$^{-/-}$ mice were stained with anti-mouse TCRγ/δ (green) and anti-mouse Foxp3 (red) to perform immunofluorescence analyses. Arrows in the merged image indicate γδTreg cells. One of the ten independent experiments is shown (F(3, 36)=10.78).

Table S1 Demographics and baseline characteristics in the full analysis set

	HC (n = 33)	RA (n = 33)	TOF (n = 13)
Mean age (years)	55.3 (11.3)	58.8 (13.8)	58.4 (10.6)
Female sex (%)	25 (75.8)	24 (72.7)	10 (76.9)
Mean duration of rheumatoid arthritis (years)	NA	10.2 (6.03)	5.6 (7.3)
DAS28 (ESR)			
Mean score	NA	6.4 (1.52)	5.7 (1.55)
Score>5.1(%)	NA	27 (81.8)	9 (69.2)
Mean C-reactive protein level (mg/L)	NA	22.9 (66.8)	36.3 (39.0)
Erythrocyte sedimentation rate (mm/h)	NA	44.8 (19.8)	42.6 (19.2)
Positive for rheumatoid factor (%)	NA	28 (84.8)	11 (84.6)
Positive for anti-CCP antibodies (%)	NA	25 (75.8)	10 (76.9)

Data are number (%) or mean (SD)

HC = healthy controls; RA = patients with rheumatoid arthritis; TOF = RA patients treated with tofacitinib 5 mg twice a day; NA = not available; DAS28(ESR) = the Disease Activity Score for 28-joint counts based on the erythrocyte sedimentation rate DAS28-4(ESR) ranges from 0 to 9.4, with higher scores indicating greater levels of disease activity (<2.6 indicates remission, ≤3.2 equals low disease activity and ＞5.1 means high disease activity); CCP=cyclic citrullinated peptide antibody.
